# Panel Doctors and Insurance Committees

**Published:** 1918-06-29

**Authors:** 


### LEGAL NOTES FOR INSTITUTIONAL WORKERS.

### Panel Doctors and Insurance Committees.

There have been two cases of considerable interest to the profession with regard to the position of panel doctors in relation to Insurance Committees. One of these was decided in England, the other in Scotland. In *Clement* v. *Devon Insurance Committee*, 144 L.T.J. 51, the question arose whether a panel doctor had submitted to arbitration. By a clause of an agreement for the payment of capitation fees, the doctor had agreed with the Insurance Committee that any dispute arising between the Committee and himself relating to the construction of the agreement or the rights and liabilities of the Committee or the practitioner should "be referred to the Commissioners." By Reg. 51 of the Medical Benefit Regulations (England), 1913, it is provided that "where under the provisions of these regulations or of any agreement made between the Committee and a practitioner on the panel . . . any question arising between the Committee and the practitioner . . . is referred, or any appeal from a decision of the Committee is made, to the Commissioners, the Commissioners shall determine such question or appeal in such manner as they think fit, and if in the opinion of the Commissioners a hearing is required, they may authorise any two or more of the Commissioners to hear and determine such question or appeal, and any decision of the Commissioners shall be final and conclusive." Lord Justice Pickford thought the words "a hearing is required" pointed to an oral hearing with witnesses, while Lord Justice Bankes thought that the regulation gave the Commissioners power to decide disputes upon documentary evidence without hearing witnesses. It is to be noted that neither the clause of the agreement nor the regulation has any direct reference to arbitration or the Arbitration Act, 1889, unless it is contained in the word "referred," and the plaintiff, who had brought an action against the Committee for capitation fees, which Mr. Justice Rowlatt refused to stay, contended on that ground, and on the ground that the procedure indicated by the regulation and the procedure under the Arbitration Act were substantially different, that he had not submitted to arbitration. Lords Justices Pickford and Bankes, and Mr. Justice Sargant, after hearing the arguments, reversed the order of Mr. Justice Rowlatt, and decided that the dispute ought to be referred to the Commissioners.

The Scottish case, *Mitchell* v. *The Insurance Committee of Aberdeen*, 1918, 1 L.T.J. 316, decides quite a few points of practical importance with regard to the procedure to be followed by a doctor in pursuing his remedies under the National Insurance Act. In the first place it decides that an Insurance Committee comes within the scope of the Public Authorities Protection Act of 1893, which precludes action being raised against them after the expiry of six months from the date of the cause of action occurring. In the next place the case emphasises the necessity of a doctor who feels aggrieved by the action of the Insurance Authorities to follow out the statutory procedure. In this case a doctor had been surcharged by the Insurance Committee in respect of over-prescribing. Instead of appealing to the Insurance Commissioners he took proceedings at common law to have the action of the Insurance Committee cut down on the ground of irregularity. His action was held incompetent, it being pointed out that his proper course was to appeal to the Insurance Commissioners. The resources of the statute—*i.e.* of the National Insurance Act—were intended to be exhausted before recourse could be made to Courts of Law. Lastly, the main point in the case was with regard to the right of surcharge. The surcharge made amounted to the large sum of £244. It was held that the Insurance Committee were entitled to deduct the amount of the surcharge from any sums that might be due to the doctor, and not merely from sums due in respect of the year in which the excessive prescribing took place.

